# Verification and Validation of SARS-CoV-2 Assay Performance on the Abbott *m*2000 and Alinity *m* Systems

**DOI:** 10.1128/JCM.03119-20

**Published:** 2021-04-20

**Authors:** Julie W. Hirschhorn, April Kegl, Tanisha Dickerson, W. Bailey Glen, Gang Xu, Jay Alden, Frederick S. Nolte

**Affiliations:** a Department of Pathology and Laboratory Medicine, Medical University of South Carolina, Charleston, South Carolina, USA; St. Jude Children’s Research Hospital

**Keywords:** COVID-19, RNA detection, real-time PCR, SARS-CoV-2

## Abstract

We verified the analytical performance of the Abbott RealTi*m*e SARS-CoV-2 assay on the *m*2000 system and compared its clinical performance to the CDC 2019-nCoV real-time PCR diagnostic panel and the Thermo Fisher TaqPath RT-PCR COVID-19 kit. We also performed a bridging study comparing the RealTi*m*e SARS-CoV-2 assay with the new Abbott Alinity *m* SARS-CoV-2 assay. A number of standards, reference materials, and commercially available controls were used for the analytical verification to confirm the limit of detection, linearity, and reproducibility. We used nasopharyngeal (NP) swab specimens collected in saline for the clinical verification and bridging studies. Overall, we found 91.2% positive percent agreement (PPA; 95% confidence interval [CI] = 76.2 to 98.14%) and a 100% negative percent agreement (NPA; 95% CI = 97.97 to 100%) between the results of the RealTi*m*e SARS-CoV-2 and CDC tests with 217 NP specimens (*P* = 0.13). We found a PPA of 100% (95% CI = 90.26 to 100%) and an NPA of 95.15% (95% CI = 83.47 to 99.4%) between the results of the RealTi*m*e and TaqPath tests with 77 NP specimens (*P* = 0.24). Finally, we tested 203 NP swab specimens for SARS-CoV-2 on the *m*2000 on the Alinity *m* systems. The PPA and NPA were 92.2% (95% CI = 85.3 to 96.59%) and 92% (95% CI = 84.8 to 96.5%), respectively (*P* = 0.4). Although cycle number (Cn) values obtained for the concordant positive samples were highly correlated (*R*^2^ = 0.95), the Cn values were on average 14.14 higher on the Alinity *m* system due to the unread cycles with the RealTi*m*e SARS-CoV-2 assay.

## Abstract (precis)

We verified the analytical performance of the Abbott RealTi*m*e SARS-CoV-2 assay on the *m*2000 system and compared its clinical performance to the CDC 2019-nCoV real-time PCR diagnostic panel and the Thermo Fisher TaqPath RT-PCR COVID-19 kit. We also performed a bridging study comparing the RealTi*m*e SARS-CoV-2 assay with the new Abbott Alinity *m* SARS-CoV-2 assay.

## INTRODUCTION

As SARS-CoV-2 continues to spread worldwide, diagnostic testing and contact tracing have become essential strategies for infection control with the goals of slowing the spread of COVID-19, minimizing the strain on health care resources, and guiding public health policy (1). While early testing efforts in the United States focused on individuals at high risk of infection, such as frontline health care providers, diagnostic testing has expanded in several states, and in some areas it is being used for surveillance in individuals who have had contact with infected individuals but who have not yet developed symptoms (2). To meet the need for expanded testing, several high-throughput molecular assays to detect SARS-CoV-2 have been approved for used by the U.S. Food and Drug Administration (FDA) under emergency use authorization (EUA). As of 3 November 2020, 189 molecular tests from test kit manufacturers and commercial laboratories and 34 molecular laboratory-developed tests from high-complexity laboratories have been given EUA status (3). Robust cross-validation studies are not available for many of these tests.

Our laboratory receives specimens from collection sites throughout the state of South Carolina for SARS-CoV-2 testing. The testing demand is expected to continue to increase as the pandemic continues and testing expands to include asymptomatic individuals for a variety of applications. Automated and semiautomated molecular diagnostic testing protocols have allowed us to perform rapid and accurate SARS-CoV-2 testing to meet this growing need.

In March 2020, Abbott Molecular (Des Plaines, IL) developed and received EUA approval for the RealT*im*e SARS-CoV-2 assay, a real-time PCR test to detect viral RNA in nasopharyngeal (NP) and oropharyngeal swabs in individuals with suspected COVID-19. The RealTi*m*e SARS-CoV-2 assay is run on the automated *m*2000 system, which is currently in place in several central labs and hospital-based labs worldwide. Abbott also received EUA for a SARS-CoV-2 assay run on its recently FDA-cleared automated assay system, the Alinity *m*. In this study, we verified the analytical performance of the RealTi*m*e SARS-CoV-2 assay on the *m*2000 system and compared its clinical performance to the CDC 2019-nCoV real-time PCR diagnostic panel and the Thermo Fisher TaqPath RT-PCR COVID-19 kit. We also performed a bridging study of the Alinity *m* SARS-CoV-2 assay. Positive results of both assays are reported with cycle number (Cn) values which are equivalent to cycle threshold (*C_T_*) values more commonly used by other real-time PCR assays.

## MATERIALS AND METHODS

### Study site and samples.

The analytical and clinical performance of the Abbott RealTi*m*e SARS-CoV-2 assay was evaluated at the Medical University of South Carolina (MUSC) Department of Pathology and Laboratory Medicine in Charleston, South Carolina. The study protocol was considered a process improvement project and not subject to IRB review.

A total of 477 flocked NP swab specimens were collected in 3 ml of universal transport medium (UTM) or sterile saline at drive through specimen collection sites in Charleston and other cities in South Carolina. Due to shortages of UTM and other traditional transport media, we switched to saline early in the pandemic and these samples comprised the majority of the specimens included in this study. An additional 10 positive NP specimens in UTM were supplied by the South Carolina Department of Health and Environmental Control (SCDHEC) Public Health Laboratory. All samples were transported to the lab at 4°C and tested within 72 h of collection or frozen at –80°C until needed, with no more than one freeze-thaw cycle. Samples were not heat inactivated prior to testing.

### Comparator assays.

The EUA CDC 2019-nCoV real-time PCR diagnostic panel uses primers and probe sets that amplify and detect the SARS-CoV-2 N1 and N2 gene regions (2019-nCoV kit; Integrated DNA Technologies), with the human RNase P gene (RP) as the internal control, according to the instructions for use (4). Inconclusive results may be reported by the assay when only one of the two target amplifications is detected. The CDC assay was performed at three different sites: a commercial referral laboratory (Premier Medical Laboratories, Greenville, SC), the SCDHEC Public Health Laboratory (Columbia, SC), and MUSC.

As an additional comparator, we used the EUA TaqPath RT-PCR COVID-19 kit (Thermo Fisher) according to the manufacturer’s instructions for use (5). The TaqPath test uses primers and probes directed to the SARS-CoV-2 virus ORF1ab, N protein, and S protein genes. Internal control primers and probes detect bacteriophage MS2. The data were analyzed and interpreted using the Applied Biosystems COVID-19 interpretative software (Thermo Fisher). Since this test was performed in research laboratory space, our University Biosafety officer required that the specimens be heat inactivated (56°C for 30 min) before transport from the clinical laboratory.

Samples sent to the commercial reference laboratory were also tested for other respiratory pathogens with the BioFire RP assay (bioMérieux, Salt Lake City, UT).

### Abbott SARS-CoV-2 assays.

The RealTi*m*e SARS-CoV-2 assay was performed at MUSC according the EUA product insert (6). The assay uses two sets of primers to amplify regions within the highly conserved RNA-dependent RNA polymerase (RdRp) and N genes. Annealing of fluorescent probes targeted to the amplified viral sequences indicates a positive test. The RealTi*m*e SARS-CoV-2 assay was run on three automated *m*2000 systems, each of which can produce up to 94 patient results/batch and produce up to 188 patient results in 8 h and 470 patient results/day if the laboratory operates with three shifts.

The Alinity *m* SARS-CoV-2 assay is run on the Abbott Alinity *m* system (Abbott Molecular, Des Plaines, IL), a fully automated, continuous, random-access molecular diagnostic analyzer using real-time PCR and ReadiFlex technology (7). It uses the same sample types, same controls, and same viral and internal control genes as the RealTi*m*e SARS-CoV-2 assay performed on the *m*2000 analyzer. The Alinity *m* has a processing capacity of 300 samples in ∼8 h and a time to first result of <2 h. It can produce up to 1,080 patient results if run over three shifts. It has an amplification reagent capacity of 20 reagent packs that can be stored on-board for 30 days.

### Reference materials.

A number of standards, reference materials, and commercially available controls were used in the analytical verification to confirm the limit of detection (LOD), linearity, and reproducibility. Purified genomic RNA from SARS-CoV-2 strain USA_WA1/2020 grown in Vero E6 cells was provided by the World Reference Center for Emerging Viruses and Arboviruses, University of Texas Medical Branch (UTMB), Galveston TX. It contained approximately 6 × 10^6^ PFU/μl or approximately 1 × 10^11^ genome equivalents/ml, based on the average of estimates by UTMB and MUSC determined by digital PCR (dPCR).

Exact Dx SARS-CoV-2 standard material consisted of E, N, S, ORF1, and RdRp gene RNA transcripts at 200,000 copies/ml and human gDNA at 75,000 copies/ml in synthetic base matrix. In addition, we used the Seracare AccuPlex SARS-CoV-2 reference material kit, replication-deficient virus containing ORF1a, RdRp, S, E, and N gene regions from SARS-CoV-2 reference material (4,162 copies/ml), and an AccuPlex SARS-CoV-2 verification panel (10^3^, 10^4^, and 10^5^ copies/ml). Quantities were assigned by the manufacturer by dPCR.

We participated in a proficiency testing program, the INSTAND Extra EQA scheme “Virus genome Detection-SARS-CoV-2.” This provided six samples of dilutions of inactivated virus-infected cell lysates, as follows: 1:10^3^, 1:10^4^, 1:10^5^, and 1:10^6^ dilutions of SARS-CoV-2 and 1:2,500 dilutions of CoV-OC43 and CoV-229E. Lysates of noninfected cells were also included as a negative control. Thirty-four of the participant laboratories quantified the amount of SARS-CoV-2 RNA in samples, and mean values were included in the participant summary report as 4.41, 5.34, 6.34, and 7.24 log copies/ml in reverse order of dilution, respectively (8).

### Statistical methods.

Analyze-It standard edition software, v3.76.1 (Leeds, UK), was used for all statistical analyses.

## RESULTS

### Analytical performance of the RealTi*m*e SARS-CoV-2 assay on the *m*2000.

**FIG 1:**
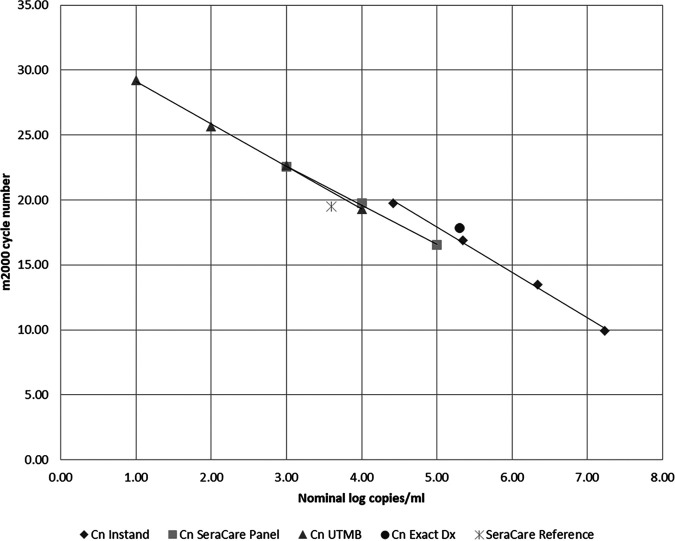
Results obtained on the RealTi*m*e SARS-CoV-2 assay on the *m*2000 system with reference materials obtained from UTMB-Galveston, SeraCare, Exact Dx, and the INSTAND EQA program. Trend lines for materials prepared as dilution series are also shown.

Initial attempts to dilute UTMB genomic RNA in UTM led to poor recovery even at the highest concentrations tested, probably as a result of rapid degradation of the naked RNA in UTM. However, the same dilutions of genomic RNA made in saline showed excellent recovery down to as low as 10 copies/ml in our initial range-finding experiments for the LOD (Fig. 1). Four replicates at each at nominal concentration of 1, 2, 3, and 4 log copies/ml were tested on two different *m*2000 instruments. The results were reproducible with a percent coefficient of variation (%CV) ranging from 1.3 to 4.7%, and the assay response was linear to the concentration range (*R*^2^ = 0.99). Figure 1 also shows results obtained with the reference material from SeraCare, Exact Dx, and the INSTAND EQA program. The trend lines for these materials prepared as dilution series are also shown and were very similar. Naked genomic RNA, naked RNA transcripts, RNA transcripts in a recombinant virus capsid, and an inactivated SARS-CoV-2-infected cell lysate behaved similarly with the RealTi*m*e SARS-CoV-2 assay, and there was excellent correlation of the Cn values with the labeled concentrations as determined by dPCR by the manufacturers across the different reference materials tested. Although the assay is a qualitative test, our results suggest that its response is linear over at least a 6-log range and that the Cn values could be used to estimate SARS-CoV-2 viral burden in clinical samples.

Although inactivated SARS-CoV-2 virus suspensions are likely the most commutable reference materials, they were not available when we performed our initial verification studies. We chose the SeraCare reference panel material to confirm the LOD of the assay because it is superior to “naked” RNA; the viral protein coat protects the RNA from degradation when added to clinical samples, and it is fully extractable. To confirm the manufacturer’s LOD claim of 100 copies/ml, we diluted SeraCare panel member 1 (1,000 copies/ml) 1:10 in saline and tested it 20 times. All 20 replicates were detected with a mean Cn of 26.73, a range of 24.49 to 29.71, a standard deviation of 1.35, and a %CV of 5.2%.

### Analytical performance of the Alinity *m* SARS-CoV-2 assay.

We confirmed the manufacturer’s LOD claim of 100 copies/ml by diluting the SeraCare AccuPlex verification panel member with a nominal concentration of 1,000 copies to 400, 200, 100, 50, and 25 copies/ml in normal saline. The number of replicates tested and the number and % positive at each concentration tested, as well as the average Cn and standard deviation, are shown in Table 1. We confirmed the manufacturer’s stated LOD of 100 copies/ml with the standard deviation of Cn values ranging from 0.83 to 1.31. We also detected 90% of the samples with 50 copies/ml. The Alinity *m* SARS-CoV-2 assay had the same LOD as the RealTi*m*e SARS-CoV-2 assay run on *m*2000 with similar imprecision of Cn values at the LOD.

**TABLE 1:** Limit of detection and reproducibility of the EUA Alinity *m* SARS-CoV-2 assay

Copies/ml	No. positive/no. tested (%)	Avg Cn	SD
400	10/10 (100)	37.52	1.08
200	10/10 (100)	38.87	0.83
100	19/20 (95)	39.78	1.31
50	9/10 (90)	40.20	1.20
25	2/10 (20)	39.05	NA

### Clinical verification of the RealTi*m*e SARS-CoV-2 assay on the m2000.

We tested 183 residual NP samples collected on March 12 to 17, 2020, from symptomatic patients and sent to a commercial referral lab that used the CDC test for SARS-CoV-2 RNA detection. The positive percent agreement (PPA) was only 75% with three apparent false negatives by the RealTi*m*e SARS-CoV-2 assay. The N1/N2 *C_T_* values in the CDC test for these false negatives were 33/35, 32/32, and 38/39. All the concordant positives had N1/N2 *C_T_* values of ≤25. The three inconclusive CDC test results were negative by the RealTi*m*e SARS-CoV-2 assay. The NPA was 100%.

Since we were unable to resolve the three discrepant results with the referral lab, we obtained ten randomly selected positive specimens that were tested by the SCDHEC Public Health Laboratory with the CDC test (*C_T_* values were not provided). All ten tested positive with the RealTi*m*e SARS-CoV-2 assay (PPA 100%), with Cn values ranging from 3.24 to 22.8.

In addition, we performed the CDC EUA test locally for 12 previously positive and 12 previously negative NP swab specimens tested with the RealTi*m*e SARS-CoV-2 assay. Both the PPA and the NPA were 100%. The average *C_T_* values of the N-target region in the CDC test and the target Cn values in the RealTi*m*e SARS-CoV-2 assay were highly correlated (*R*^2^ = 0.98), but the Cn values for RealTi*m*e SARS-CoV-2 assay were much lower than the *C_T_* values for the CDC tests (data not shown). This is due to the first ten unread cycles with the RealTi*m*e SARS-CoV-2 assay.

Overall, we found a 91.2% PPA (95% CI = 76.2 to 98.14%) and a 100% NPA (95% CI = 97.97% to 100%) when comparing the results of the RealTi*m*e and CDC SARS-CoV-2 assays performed in three different laboratories across 217 symptomatic NP specimens (Table 2). The McNemar chi-square test statistic was 1.33, with a one-sided *P* value of 0.13. Considering that we found 100% PPA with the CDC test results when performed at the SCDHEC Public Health Laboratory and at our laboratory, and the excellent analytical sensitivity of the RealTi*m*e SARS-CoV-2 assay, the three discordant positive results with samples tested at the commercial referral lab were likely false-positive CDC test results and not false-negative RealTi*m*e SARS-CoV-2 assay results. However, they were counted as false-negative RealTi*m*e SARS-CoV-2 assay results in the data analysis since we had no way to resolve these discrepant results.

**TABLE 2:** Comparison of results from the EUA CDC 2019-nCoV real-time PCR diagnosis panel and Abbott RealTi*m*e SARS-CoV-2 assay

*m*2000 result	No. of CDC results			Total no.
	Positive	Inconclusive	Negative	
Positive	31	0	0	31
Negative	3	3	180	186
Total	34	3	180	217

The initial 183 NP specimens sent to the referral lab for SARS-CoV-2 testing were also tested locally with the BioFire RP assay to test for cross-reactions with other respiratory pathogens. In the 168 specimens that were negative for SARS-CoV-2 RNA by both tests (concordant negatives) other respiratory pathogens were found in 71 (42.2%) as follows: 27, rhinovirus (RV)/enterovirus (EV); 18 seasonal CoVs (13 NL63, 2 HKU 1, 2 OC43, and 1 229E); 1 CoV OC43 and RV/EV; 11 influenza A virus; 5 metapneumovirus; 2 metapneumovirus and RV/EV; 3 influenza B virus; and 1 each of Bordetella pertussis, Mycoplasma pneumoniae, parainfluenza virus 4, and respiratory syncytial virus. The RealTi*m*e SARS-CoV-2 assay showed no cross-reaction with the seasonal CoVs as well as other common respiratory pathogens.

In the nine specimens with concordant positive results for SARS-CoV-2, no other respiratory pathogen was detected. However, of the three CDC assay inconclusive/RealTi*m*e SARS-CoV-2 assay-negative specimens, influenza A virus was found in one specimen and metapneumovirus was found in another. Of the three CDC assay-positive/RealTi*m*e SARS-CoV-2 assay-negative specimens, RV/EV was found in one specimen.

Early in the pandemic, from 12 to 17 March 2020, RV/EV and seasonal CoV were detected more frequently than SARS-CoV-2; we also detected as much influenza A virus as SARS-CoV-2 during that time period.

We also compared the results of the RealTi*m*e SARS-CoV-2 assay on the *m*2000 with results obtained with the TaqPath RT-PCR COVID-19 Combo kit using 38 previously positive and 39 previously negative residual NP swab samples originally tested on the *m*2000 system (Table 3). We found a PPA of 100% (95% CI = 90.26% to 100%) and an NPA of 95.15% (95% CI = 83.47 to 99.4%). The McNemar chi-square test statistic was 0.5 with a one-sided *P* value of 0.24. The two samples not detected by the TaqPath test had Cn values of 27.1 and 27.5, which correspond to the values seen at the LOD of the RealTi*m*e SARS-CoV-2 assay. Since the LODs of the RealTi*m*e SARS-CoV-2 assay and TaqPath test were 100 and 2,000 copies/ml (as determined by limiting dilutions of the TaqPath positive control), respectively, it was expected that the TaqPath test would not detect these two low-positive samples. In addition, the specimens analyzed by the TaqPath test were heat inactivated, which has been shown to degrade viral RNA as reflected by increased *C_T_* values (9).

**TABLE 3:** Comparison of results from the EUA TaqPath RT-PCR COVID-19 kit and RealTi*m*e SARS-CoV-2 assay

*m*2000 result	No. of TaqPath results		Total no.
	Positive	Negative	
Positive	36	2	38
Negative	0	39	39
Total	36	41	77

**FIG 2:**
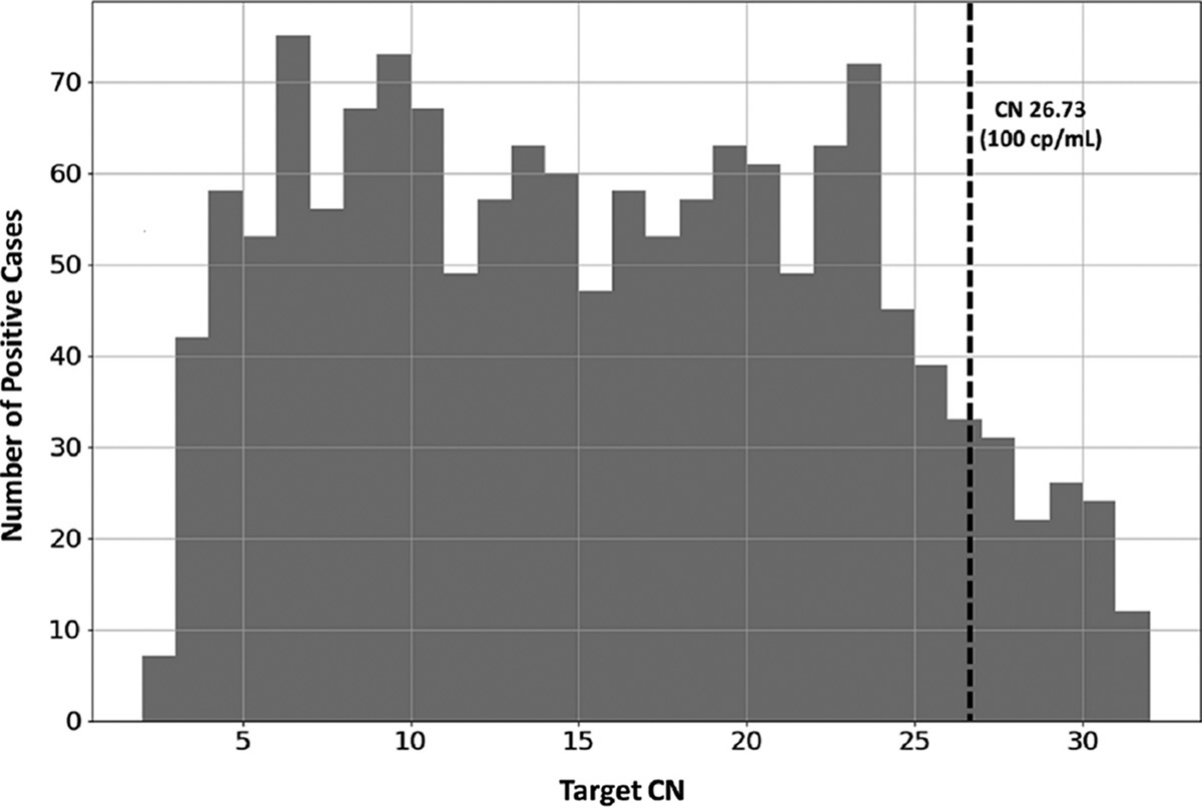
Distribution of Cn values obtained with the first 1,488 positive clinical specimens samples run with the RealTi*m*e SARS-CoV-2 assay on *m*2000 system. The dashed line is at the Cn value that corresponds to a viral load of 100 copies/ml.

Figure 2 shows the frequency distribution of Cn values obtained with the first 1,488 positive clinical specimens tested between 23 March and 31 May 2020 on the RealTi*m*e SARS-CoV-2 assay. The values ranged from 2 to 33. Of note, 8% of specimens had very low viral burdens with Cn values greater than the Cn value at the 100 copy/ml LOD (Cn 26.73).

Of note, as the pandemic progressed and our criteria for testing opened up to asymptomatic individuals for preprocedure testing and other indications, the distribution of the Cn values were more bimodal during its first peak in July 2020 and then shifted to more individuals with higher Cn values after our first wave (data not shown).

### Clinical verification of the Alinity *m* SARS-CoV-2 assay.

We tested 203 residual clinical NP swab specimens that had been previously tested with the RealTi*m*e SARS-CoV-2 assay on the *m*2000 (103 positives and 100 negatives) with the Alinity *m* SARS-CoV-2 assay run on the Alinity *m* instrument. The specimens were collected from both symptomatic and asymptomatic individuals suspected of SARS-CoV-2 infection and stored at –80°C until used in this study. We compared both the qualitative results and Cn values obtained with each assay on each instrument.

Table 4 shows the qualitative results obtained with all clinical specimens, along with the PPA and NPA and the 95% CI values for these estimates. The PPA and NPA were 92.2% (95% CI = 85.3 to 96.59%) and 92% (95% CI = 84.8 to 96.5%), respectively. The McNemar chi-square test statistic was 0.063, with a one-sided *P* value of 0.4. The average Cn values for the RealTi*m*e SARS-CoV-2 assay positive/Alinity *m* SARS-CoV-2 assay negative specimens was 29.6 (range, 27.8 to 30.64) and for the RealTi*m*e SARS-CoV-2 assay-negative/Alinity *m* SARS-CoV-2 assay-positive specimens was 31.1 (range, 17.21 to 40.99). Although there was insufficient residual material to retest the discordant specimens by the same or a different method, these samples, with few exceptions, likely had low viral loads that would be expected to give inconsistent results since they were near the LODs for both tests.

**TABLE 4:** Comparison of results from the EUA RealTi*m*e SARS-CoV-2 assay on the *m*2000 and Alinity *m* SARS-CoV-2 assay

Alinity *m* result	No. of *m*2000 results		Total no.
	Positive	Negative	
Positive	95	8	103
Negative	8	92	100
Total	103	100	203

**FIG 3:**
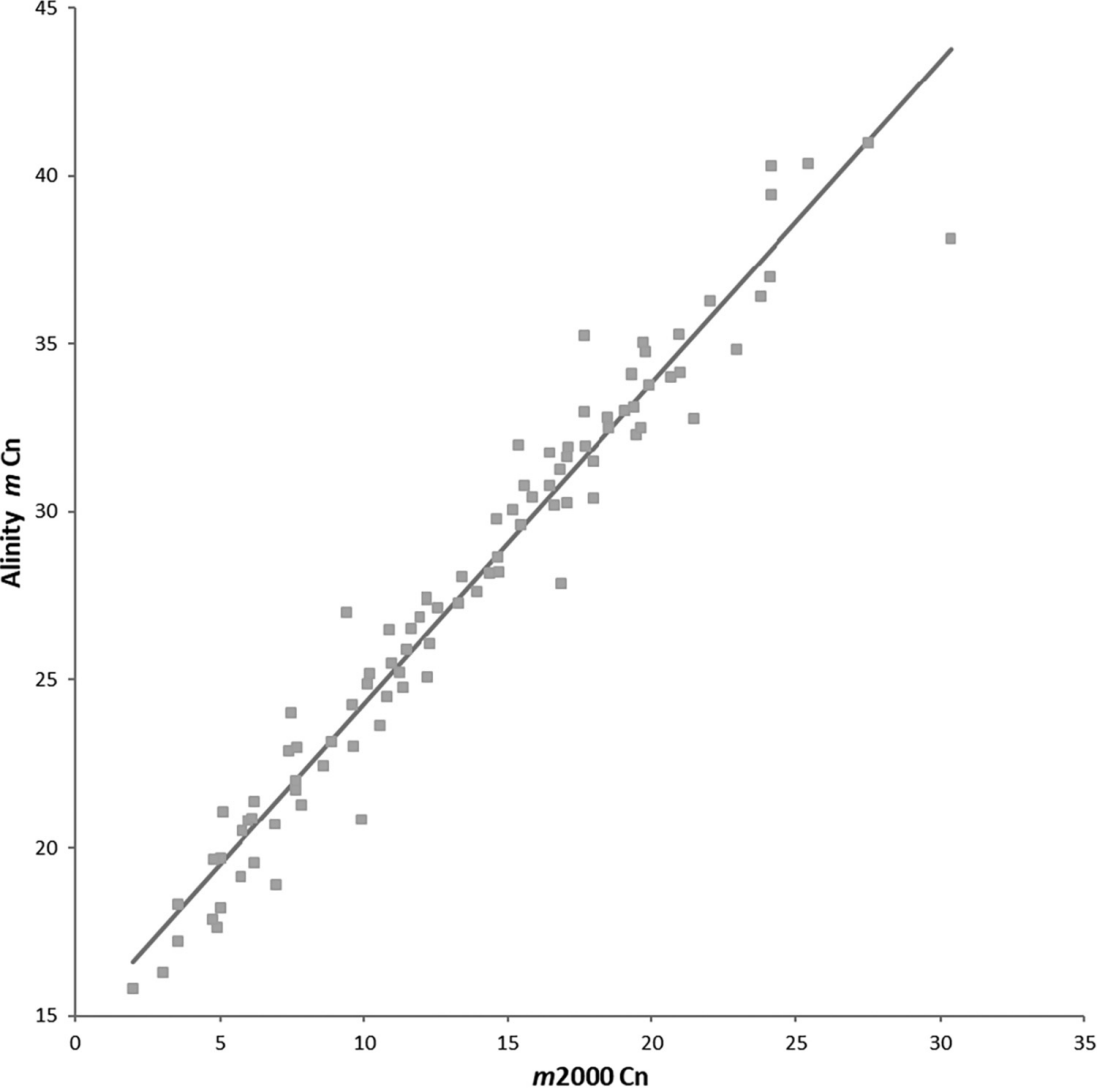
Correlation of the Cn values obtained for the 95 concordant positive samples tested with the RealTi*m*e SARS-CoV-2 assay on the *m*2000 system and the Alinity *m* SARS-CoV-2 assay. The results were highly correlated, with an *R*^2^ value of 0.95 and the fit line described by the equation: Alinity *m* Cn = 0.96 *m*2000 Cn + 14.72.

**FIG 4:**
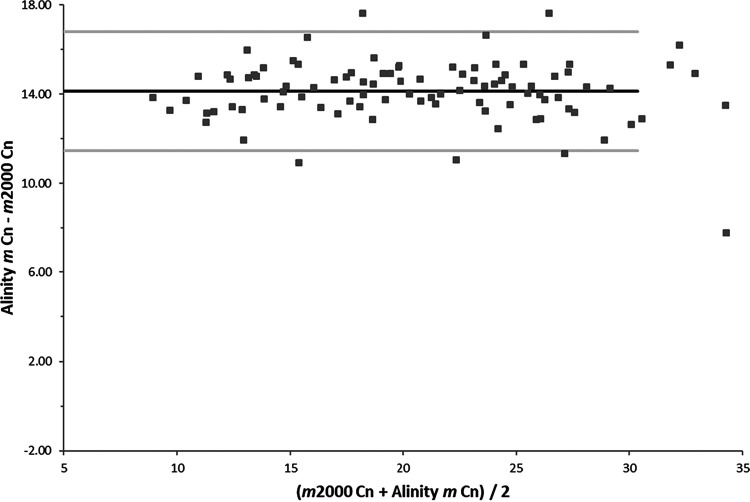
Agreement of the Cn values obtained for the 95 concordant positive samples tested with the RealTi*m*e SARS-CoV-2 assay on the *m*2000 and the Alinity *m* SARS-CoV-2 assay. The Cn values had a mean difference of 14.14 (black line) with 95% lower and upper limits of agreement of 11.47 and 16.81, respectively (gray lines).

The correlation and agreement of the Cn values obtained for the 95 concordant samples are shown in the Fig. 3 and 4, respectively. The results were highly correlated, with an *R*^2^ value of 0.95 and the fit line described by the following equation: Alinity *m* Cn = 0.96 *m*2000 Cn + 14.72. However, the Cn values had a mean difference of 14.14, with 95% lower and upper limits of agreement of 11.47 and 16.81, respectively. The difference is in part due to the first 10 unread cycles on the *m*2000 system.

## DISCUSSION

In this study, we verified the analytical and clinical performance of the RealTi*m*e SARS-CoV-2 assay on the *m*2000 system and provide the first report of the performance characteristics of the new Alinity *m* SARS-CoV-2 assay. Analytical verification of SARS-CoV-2 tests was hampered early in the pandemic by the lack appropriate reference materials. Initially, only genomic RNA or RNA transcripts were available. We found that naked RNA preparations were unstable in viral transport medium (VTM) and UTM, so we used sterile saline to perform the limiting dilution experiments with genomic RNA. We also switched to sterile saline as the transport medium for clinical specimens early in the pandemic, since VTM and UTM were unavailable due to supply chain issues. We continue to manufacture our own collection kits consisting of 3 ml of saline in sterile tube in a 15-ml conical centrifuge tube and flocked mini-tip swab to mitigate the supply chain issues with commercially available transport media.

We found that naked genomic RNA, naked RNA transcripts, RNA transcripts in a recombinant virus capsid, and an inactivated SARS-CoV-2-infected cell lysate behaved similarly in the RealTi*m*e SARS-CoV-2 assay and that there was excellent correlation of the Cn values with the labeled concentrations determined by dPCR across the different reference materials tested. We felt it was important to use multiple reference materials since there are no internationally recognized SARS-CoV-2 calibration and validation materials. The SeraCare AccuPlex SARS-CoV-2 reference material kit was used to determine the LOD of both the RealTi*m*e and the Alinity *m* SARS-CoV-2 assays because it provides in-process monitoring of all steps of the assay and was readily available. We confirmed the manufacturer’s LOD claim of 100 copies/ml for both the RealTi*m*e SARS-CoV-2 assay on the *m*2000 and the Alinity *m* SARS-CoV-2 assay run on the Alinity *m* system. The LODs for the two comparator assays used in this study, the EUA CDC and TaqPath assays, were substantially higher, at 1,000 and 2,000 copies/ml, respectively (data not shown).

We found that 8% of positive specimens initially tested on the *m*2000 had very low viral burdens with Cn values greater than the Cn value at the 100-copies/ml LOD. The reproducibility of positive specimens with high Cn values was not determined but is under investigation.

The LOD of 100 copies/ml is in stark contrast to the 5,400 nucleic acid amplification test (NAAT) detectable units (NDU)/ml reported by the FDA when the FDA SARS-CoV-2 reference panel was used (10). The FDA material used in the panel is described as a heat-inactivated strain (2019-nCoV/USA/Wa1/2020) in cell culture medium with a labeled concentration of approximately 1.8 × 10^8^ RNA NDU/ml by RT-qPCR. However, the FDA did not specify how the concentration was assigned or how NDU/ml correlates with copies/ml. This discrepancy between the LOD of the same assay using different reference materials highlights the need for harmonized calibration and validation materials to support successful deployment of reliable tests with transparent knowledge of test performance.

We assessed the clinical performance of the RealTi*m*e SARS-CoV-2 assay by parallel testing of NP swab specimens with the CDC and TaqPath EUA assays. We found excellent PPA and NPA between the RealTi*m*e SARS-CoV-2 assay and these comparator assays with no significant difference in performance. Most of the discrepant results between the different assays were obtained with specimens close to the LODs of the assays, in which the RNA may have degraded during storage or after freeze-thawing or heat inactivation.

We also performed a bridging study comparing the Abbott SARS-CoV-2 assays designed for the *m*2000 and Alinity *m* instruments. The assays had the same LODs on their respective systems and the results with clinical specimens showed 92.2% PPA and 92% NPA (*P* = 0.4). Discrepant samples generally were near the LOD for both assays. However, there was a significant difference in the Cn values reported by the two instruments, with the Alinity *m* instrument Cn values being on average 14.14 cycles higher, due, in part, to the *m*2000 instrument not reading the first 10 cycles. We currently do not report Cn values to clinicians, but with growing interest in using such values as a prognostic indicator and to establish indicators for active infection and transmissibility, there is increasing pressure on clinical laboratories to do so (11). In laboratories like ours that use both instruments, we would need to convert the Cn values to the same scale prior to reporting to prevent confusion among clinicians. In addition, we use two other real-time PCR assays from Cepheid and Thermo Fisher and two isothermal amplification assays from Hologic and Abbott (ID Now). It would be a very complicated exercise to normalize the crossing values from the four real-time PCR assays and then include them in the report with the qualitative results. In addition, no crossing values are generated by the Hologic and ID Now assays since they are isothermal amplification methods.

The Alinity *m* instrument offers several advantages over the *m*2000 instrument, including random access, a more rapid analysis time, less hands-on time, STAT functionality for urgent results, and higher throughput. A single Alinity *m* instrument can produce as many SARS-CoV-2 assay results as 3 *m*2000 instruments. Currently, our laboratory has three *m*2000 and two Alinity *m* instruments that can be operated over three 8-h shifts. This gives us a maximum daily capacity of 3,268 tests, with 1,128 performed on the *m*2000 and 2,160 on the Alinity *m* instruments. Although our health care system employs several other molecular tests for SARS-CoV-2, the Abbott instruments provide our largest test capacity and have allowed us to keep up with the unprecedented demand for testing. We performed 168,624 SARS-CoV-2 tests on the Abbott systems from 23 March through 3 November 2020 for patients in our health care system and throughout South Carolina.

One strength of our study is that we used of several sets of samples from a diverse population. Samples were obtained from various sample collection sites throughout the state, from both symptomatic and asymptomatic individuals, as well as perioperative samples collected from patients prior to scheduled procedures. We also used two different comparator assays as part of the clinical verification.

By testing our first group of verification specimens with the BioFire RP assay, our studies also provide some insight into local respiratory virus epidemiology in the early days of the pandemic in South Carolina. We found more infections with RV/EV, seasonal CoV, and influenza A virus than with SARS-CoV-2 in mid-March, highlighting the importance that other respiratory viruses played early in the pandemic. What is remarkable is the data moving forward this winter. The pandemic has completely changed the local epidemiology of respiratory viral infections. Rhinovirus/enterovirus detections have remained relatively constant throughout, but we have seen very few detections of adenovirus, parainfluenza viruses, and influenza B virus. None of the other viruses included in the BioFire panel have been detected this fall and winter. Particularly notable is the complete absence of seasonal coronavirus detections.

Our results with the RealTi*m*e SARS-CoV-2 assay on the *m*2000 system are similar to those reported Degli-Angeli et al. (12). However, we tested many more clinical specimens, included an additional comparator assay, and used a variety of reference materials to confirm its analytical performance.

Limitations of our study include that the vast majority of samples used for both the analytical and clinical verification of the Abbott tests used saline and the sample matrix. We did not adequately compare saline to more traditional collection transport media, such as UTM or VTM, due to supply chain issues, but previous studies indicate that saline performs comparably to other media for collection and transport of specimens (13, 14). We also did not verify the test performance characteristics for specimens other than NP swabs. In addition, we did not heat inactivate specimens at 56°C for 30 min prior to testing on the Abbott instruments because they provide adequate protection for operators against aerosol and droplet exposure. Thermal inactivation may degrade the single-stranded RNA target and cause false negatives in real-time PCRs, so our results may differ from laboratories that choose to do so (9).

In conclusion, we validated the analytical performance of the RealTi*m*e SARS-CoV-2 assay and compared its clinical performance to the EUA CDC and TaqPath tests using a real-world set of clinical samples. We demonstrated that the performance characteristics of the RealTi*m*e SARS-CoV-2 assay run on the *m*2000 and the Alinity *m* SARS-CoV-2 assay run on the Alinity *m* system were similar. The potential to ramp up SARS-CoV-2 testing using the automated *m*2000 system and to add to our lab capacity with the random-access, automated Alinity *m* system will support our efforts to expand testing with the goal of reducing the spread of COVID-19.
